# Book Reviews

**Published:** 1915-10

**Authors:** 


### BOOK REVIEWS

Books mentioned may be inspected at and ordered through this office. So far as possible, books received in any month will be reviewed in the issue of the second month following.

**Fractures and Dislocations.** Miller E. Preston, A. B., M. D., Denver; C. V. Mosby Co., St. Louis. 813 pages, 860 illustrations, $6.50.

This work is divided according to anatomic regions and includes also the following chapters on special subjects: terms and definitions; use of the X-Ray, by H. G. Stover of Denver; open treatment of fractures, bone transplantation, treatment of compound fractures and dislocations. It is thus adapted both to systematic study and to ready reference in an emergency. Diagnosis, therapeutics and other points of practical and theoretic interest are considered in detail under each regional subdivision. Dr. Stover deserves credit as joint author while all of Dr. Albee's work on autogenic bone grafting is included. We need not dwell on the value of the work to the surgeon. Practical experience, and thorough study of literature mark the authorship while the publishers have facilitated the work of the reader by clearness of type and illustrations, neatness of arrangement, etc. What we wish to emphasize, is the limb and life saving importance of the work to the occasional surgeon, in other words, the general practitioner. No book can insure personal skill, judgement and dexterity but this book can, at least, put the man, confronted with a surgical emergency, almost immediately in possession of all available information as to the particular surgical problem with which he has to deal.

**Operative Gynecology.** Harry Sturgeon Crossen, M. D., St. Louis. C. V. Mosby Co., St. Louis. 670 pages, 770 original illustrations, $7.70.

This volume deals exclusively with the operative treatment of the female generative organs, excluding adjacent organs and well understood principles of surgical technic in general. Having thus limited his field of authorship, the fullest discussion of the problems arising within it, but without verbal prolixity, has been the aim of the author. It should be clearly understood that both abdominal section and other forms of operation, major and minor and both destructive and conservative operations are included and that the external genitals and various special problems are included. Medicolegal points are also discussed. So far as is humanly possible, the author has placed the reader in the position of the trained surgical gynecologist, for each problem.

---

**X-Rays: How to Produce and Interpret Them.** Harold Mowatt, M. D., Temporary Lt., British Army Medical Corps, Meebut Indian General Hospital. Published by Oxford University Press, Am. Branch, 35 W. 32 St., N. Y. 204 pages, copiously illustrated, $3.00.

The title of the book give a good idea of the practical way in which the subject is treated, and also indicates that the main consideration is diagnostic rather than therapeutic. As a curative measure, X-Rays are a two-edged sword and while much valuable work is being done along therapeutic lines, the time has not arrived at which positive statements of possibilities and limitations can be made. It is not so generally understood that the diagnostic use of the X-Rays is beset with the same dangers and only slightly less direct. Not only is the wrongly operated X-Ray tube dangerous to operator and patient, but the misinterpretation of shadows, whether observed by the eye directly or after recording them on a photographic plate, subjects the patient to the same indirect dangers as a false diagnosis made by any other means. It may even be said that we are not past the false conception that by the X-Rays, we look through the body and observe conditions concealed from the naked eye. A work like the present, em bodying both scientific research and practical experience, tempered by common sense, is a great aid in making the X-Rays valuable and reliable.

---

**Simplified Infant Feeding.** Roger H. Dennett, B. S., M. D., New York. J. B. Lippincott Co., Philadelphia, 355 pages, 14 illustrations, $3.00.

An original point in the arrangement of this work is the substitution for a table of contents, of a preliminary chapter containing a synopsis of the text and thus giving a bird's-eye view of the subject. A somewhat delicate but very necessary problem is solved by appending an analysis of the proprietary foods commonly used. Indications for special diets are considered by diseases and disturbed conditions and the whole work is of a practical nature and evinces great care and much experience.

---

**A Text Book of Surgery for Students and Practitioners.** By George Emerson Brewer, A. M., M. D., Professor of Surgery, College of Physicans and Surgeons, New York; Surgical

Director, Presbyterian Hospital; Consulting Surgeon, Roosevelt Hospital, assisted by Adrian V. S. Lambert, M. D., Associate Professor of Surgery, Columbia University; Attending Surgeon, Presbyterian Hospital; and by members of the surgical teaching staff of Columbia University. Third edition, thoroughly revised and rewritten. Octavo, 1027 pages, with 500 engravings and 23 plates in colors and monochrome. Cloth, net, $5.50. Lea & Febiger, Publishers, Philadelphia and New York, 1915.

This treatise covers first the general principles of surgery and then takes up the systems and apparatuses seriatim. The colored plates are especially good. The attention paid to the thyroid gland, the thymus and status lymphaticus, the lymphatic system, mediastinum, etc., is especially valuable.

---

**Practical Materia Medica and Prescription Writing.** Oscar W. Bethea, M. D., Ph. G., F. C. S., New Orleans; F. A. Davis Co., Philadelphia.

The part on Materia Medica is arranged alphabetically by drug names. That on Prescription Writing is conservative in its advocacy of Latin and the greater use of the apothecary's system. It enters into a good many details as to labeling, preserving records, telephoning prescriptions, economy in quantity ordered, arrangement of blanks, etc., not usually considered in books. A large number of actual prescriptions are presented, mostly in script and errors in nomenclature, use of Latin, incompatibility, dosage, etc., are pointed out. This is a highly practical way of teaching correct prescription writing.

---

**Amnesia and Analgesia in Parturition (Twilight Sleep).** Alfred M. Hellman, M. A., M. D., New York; Paul B. Hoeber, N. Y. 137 pages, $1.50.

This is a careful study of a much discussed subject, presenting not only the Freiburg method but various other methods of painless labor. Case reports and statistics and a full bibliography are given. The conclusions are conservative. A novel feature of this work is that supplementary pages will be published from time to time and will be sent free to purchasers of the book, on request. Blank pages are also left for personal bibliographic notes.

---

**Cancer: Its Study and Prevention.** By Howard Canning Taylor, M. D., Gynecologist to the Roosevelt Hospital, New York; Professor of Clinical Gynecology, Columbia University; Member American Society for the Control of Cancer, etc. 12mo, 330 pages. Cloth, $2.50, net. Lea & Febiger, Publishers, Philadelphia and New York, 1915.

This work, though small in volume, shows evidence of careful study and the facts presented are supported by statistics, so far as possible. For example, the vexed question as to whether cancer is really increasing or not is not categorically answered but English-Welch statistics show that the ratios of cancer deaths to living population and to total deaths are rapidly changing so as to indicate such an increase.

---

**A Synopsis of Medical Treatment.** By George C. Shattuck, M. D., Second revised printing of the second edition, 185 pages, price $1.25. W. M. Leonard, Boston, Publisher.

This book is based on the post graduate course at the Massachusetts General Hospital. The conditions discussed are grouped under Cardiac Insufficiency, Nephritis, Acute Infections (including various respiratory conditions), Gastric and Duodenal Ulcer, (including a number of other gastro-enteric diseases), and the work also epitomizes materia medica, 30 drugs being discussed in some detail, 16 listed for occasional use and 21 for common use, about which, of course, no two persons would agree. The only criticism that we would make on such lists is that a drug—or other therapeutic method—may be used very rarely and still should be remembered as being indicated to the extent of 50, 75, or 100 per cent. in occasional instances. Moreover, the frequency of use depends largely on the personal equation and on local factors of incidence, etc. Personally, we have found it advisable to keep well in mind about 300 drugs as efficient in conditions likely to be encountered even in special practice. But there are a surprising number of "drugs," alcohol, salt, ether, chloroform, etc., kept on hand in offices that are not usually included in any attempt to list workable drugs.

---

**La Vaccination Anti-Typhoidique.** Dr. H. Mery, Paris. Librarie J. B. Bailliere et Fils, 19 Rue Hautefeuille, Paris. 95 pages, 1 F. 30.

This is one of the familiar blue-covered monographs of the series of Les Actualites Medicales (present day medicine) published by our French agents. The history of anti-typhoid vaccination is traced from the memoire of Chantemesse and Widal in 1888. Three general methods are recognized: 1 (a) By bacilli killed by heat (Wright, Chantemesse, Pasteur Institute, Pfeiffer and Kolle, Russell); 1 (b) By bacilli killed by antiseptics (Semple & Matson, Levy & Blumenthal, Vincent (multivalent); 2 By living bacilli, Besredka, Ch. Nicolle, A. Conor & Conseil). 3. By autolysats, bacillary extracts (Wassermann, Shiga & Neisser, Conradi, Bassenge & Mayer, MacFayden & Rowland, Vincena, Lumiere & Chevrotier, the last intended for internal administration). Practically, the method is limited to the use of bacilli killed by heat (Wright, Chantemesse), bacilli killed by ether (Vincent), and the living cultures (Besredka and Ch. Nicolle). The theory of immunization technique etc., are thoroughly discussed and various statistics are collated, showing the value of the procedure. This is one of the most practical treatises on the subject that has appeared, it is international in scope and deserves careful reading by the profession of this country.

---

**The Principles of Human Physiology.** New (2nd) edition By Ernest H. Starling, M. D., F. R. C. P., F. R. S., Jodrell Professor of Physiology in University College, London. Octavo, 1271 pages with 566 illustrations, including 10 in colors. Cloth, $5.00, net. Lea & Febiger, Publishers, New York and Philadelphia, 1915.

This work already has an established reputation. Physiology has, in recent years, made great progress, not only in the extension of knowledge along lines familiar to the older students but by the development of new conceptions of the phenomena of life. The following chapters deserve special attention: The mechanism of organic synthesis which materially assists the grasp of organic chemistry. The energy of molecules in solution. The properties of colloids, which partially bridge the gap between physics and chemistry. The defense of the organism against infection, and also various parts of other chapters, dealing with ferments and hormones and discussing the interrelations of the ductless glands. As might be expected, also, an increasing practicality is noted, not in the superficial sense but as the thorough understanding of the action of the various viscera in nutrition and elimination, aids the comprehension of disease and throws light on methods of diagnosis and treatment.

---

**Collected Papers From the Research Laboratories of Parke, Davis & Co., Detroit.** Compiled by Dr. E. M. Houghton, Director. This collection comprises Nos. 53 to 74, reprinted from various magazines, in uniform type and on uniform paper.

The range covered is bacteriology, the preparation of serums, pharmacology and physiology. Except by their general application to practical medicine, these papers do not bear especially on pharmacology, much less on the use of products of this well known firm. On the contrary, the work is of the highest scientific value and has been conducted in a scientific spirit without ulterior motives. The papers are exceedingly valuable and deserve careful study both by laboratory workers and those who wish to keep in touch with scientific advance along medical lines.

---

**Habits That Handicap.** Dr. Charles B. Towns, The Century Co. New York. 289 pages, $1.20.

This book covers alcohol, tobacco and the various habit forming drugs usually so considered. It discusses psychology, the need of special treatment and the function of sanatoriums. While especially intended for the laity, the book will prove interesting to the medical profession.

---

**Flies and Diarrheal Disease.** Publication No. 91, New York Association for Improving the Condition of the Poor.

The Bureau of Public Health and Hygiene of the New York Association for Improving the Condition of the Poor has issued a special publication entitled, "Flies and Diarrheal Disease," descriptive of its three months study in the homes of over a thousand infants in New York City on the relation of flies and diarrheal disease. Special attention has been given such influencing factors as dirt and artificial feeding, and their relative importance determined. A full description of the study with its important conclusions may be obtained by request from Philip S. Platt, Superintendent of the Bureau, 105 East 22d St., New York City.

